# Tantalum-carbon-integrated nanozymes as a nano-radiosensitizer for radiotherapy enhancement

**DOI:** 10.3389/fbioe.2022.1042646

**Published:** 2022-10-24

**Authors:** Rui Li, Weiheng Zhao, Tingting Wu, Aifeng Wang, Qing Li, Ying Liu, Huihua Xiong

**Affiliations:** ^1^ Department of Oncology, Tongji Hospital, Huazhong University of Science and Technology, Wuhan, China; ^2^ Department of Pharmacy, Henan Provincial People’s Hospital, Department of Pharmacy of Centeral China Fuwai Hospital, Centeral China Fuwai Hospital of Zhengzhou University, Zheng Zhou, China

**Keywords:** hypoxia, nanozyme, radiotherapy, ROS, tantalum

## Abstract

Radiotherapy (RT) plays a pivotal role in the comprehensive treatment of multiple malignant tumors, exerting its anti-tumor effects through direct induction of double-strand breaks (DSBs) or indirect induction of reactive oxygen species (ROS) production. However, RT resistance remains a therapeutic obstacle that leads to cancer recurrence and treatment failure. In this study, we synthesised a tantalum-carbon-integrated nanozyme with excellent catalase-like (CAT-like) activity and radiosensitivity by immobilising an ultrasmall tantalum nanozyme into a metal-organic framework (MOF)-derived carbon nanozyme through *in situ* reduction. The integrated tantalum nanozyme significantly increased the CAT activity of the carbon nanozyme, which promoted the production of more oxygen and increased the ROS levels. By improving hypoxia and increasing the level of ROS, more DNA DSBs occur at the cellular level, which, in turn, improves the sensitivity of RT. Moreover, tantalum–carbon-integrated nanozymes combined with RT have demonstrated notable anti-tumor activity *in vivo*. Therefore, exploiting the enzymatic activity and the effect of ROS amplification of this nanozyme has the potential to overcome resistance to RT, which may offer new horizons for nanozyme-based remedies for biomedical applications.

## 1 Introduction

Malignant tumors are refractory diseases with high morbidity and mortality rates, which seriously threatens human health (Sung et al., 2021). Radiotherapy (RT) is a commonly used treatment modality (Gong et al., 2021) that achieves locoregional tumor control mainly through direct physical damage to DNA or indirect damage from reactive oxygen species (ROS) (Schaue and McBride, 2015). However, in clinical practice, patients can develop different degrees of resistance to RT, leading to treatment failure and recurrence of metastasis, thus limiting the efficacy of RT for treatment of tumors (Makhov et al., 2018). Addressing the sensitivity of RT has become a pivotal issue in the efficacy of RT in patients with tumors (Huang and Zhou, 2020; Thielhelm et al., 2021; Li et al., 2022).

Thus far, the possible mechanisms of radiation resistance include ROS levels, tumor cell hypoxia, DNA radiation damage repair, glutathione (GSH) content, cell proliferation, cycle regulation, and the activation of related RT resistance signaling pathways (Song et al., 2017a; Xie et al., 2019; Chen et al., 2020; Suo et al., 2020; Huang et al., 2021a). The free radicals generated during RT treatment are the main source of ROS, which promote the effects of RT by acting on DNA double-strand breaks (DSBs) (Cheng et al., 2018; Sun et al., 2018; Srinivas et al., 2019; Huang and Pan, 2020). In addition, the supply of oxygen in the tumor microenvironment is a crucial factor in the killing effect of RT (Dou et al., 2018). Studies have revealed that the tumor microenvironment is in a hypoxic state, which induces large secretion of vascular production factors, chemokines, and biologically active mediators, promoting tumor progression and metastasis (Gilkes et al., 2014; Hompland et al., 2021; Kopecka et al., 2021). The radiosensitivity of cells irradiated in the presence of oxygen is approximately three times higher than that in the absence of oxygen; therefore, the radiation dose required to kill oxygen-depleted cells is significantly higher than that required to kill fully oxygenated cells (Kabakov and Yakimova, 2021; Telarovic et al., 2021). The existence of a hypoxic tumor microenvironment enables the broken single-stranded DNA structure to be repaired by moieties such as sulfhydryl groups, resulting in fewer DNA damage sites, reduced tumor cell apoptosis, and ultimately radioresistance. In contrast, RT generates a large amount of ROS, which are produced by bombarding oxygen molecules with high-energy electrons. Thus, some tumors are less sensitive to RT owing to their high levels of internal hypoxia and low oxygen content (Graham and Unger, 2018; Bolland et al., 2021).

In recent years, owing to the excellent biocompatibility and safety of nanomaterials, nanotechnology-based RT sensitisation has garnered considerable attention and has exhibited promising potential for improving the effectiveness of RT, reducing toxic side effects, and improving prognosis (Gao et al., 2017; Chen et al., 2019; Huang et al., 2021b; Sun et al., 2021). Several metal element nanoparticles with high atomic numbers exhibit RT sensitising properties (Guo et al., 2017; Feng et al., 2018; Huang et al., 2019; Lu et al., 2021; Yang et al., 2021; Yang et al., 2022). It has a high X-ray absorption capacity, which can increase the dose of precipitated RT. When the metal nanoparticles reach the tumor site, they are subjected to radioactive irradiation, and after the particles absorb radiation, various effects (such as the photoelectric and Compton effects) occur, releasing a diverse range of particles such as photoelectrons, Compton electrons, and Auger electrons, which react with organic molecules or water in cancer cells to generate a large number of free radicals, thus improving the effect of RT. In addition, nanoparticles can achieve nano-RT sensitisation by regulating the cell cycle, depleting GSH, remoulding tumor vasculature, relieving hypoxia in the tumor microenvironment, and loading chemotherapeutic drugs (Zhang et al., 2019; Lyu et al., 2020; Wang et al., 2021; Zhou et al., 2021; Gao et al., 2022). For instance, Pei Pan et al. (2022) exploited an integrated nanosystem (Bac@BNP) that enhances the sensitivity of RT by modulating the cell cycle and increasing the level of ROS. In a study by Ma et al. (2021), a bismuth nitrate-loaded cisplatin prodrug (NP@PVP) was synthesised to enhance DNA damage after RT by improving the amount of ROS production; meanwhile, cisplatin in NP@PVP can be released slowly to inhibit DNA damage repair, with temporal and spatial synchronisation.

**SCHEME 1:**
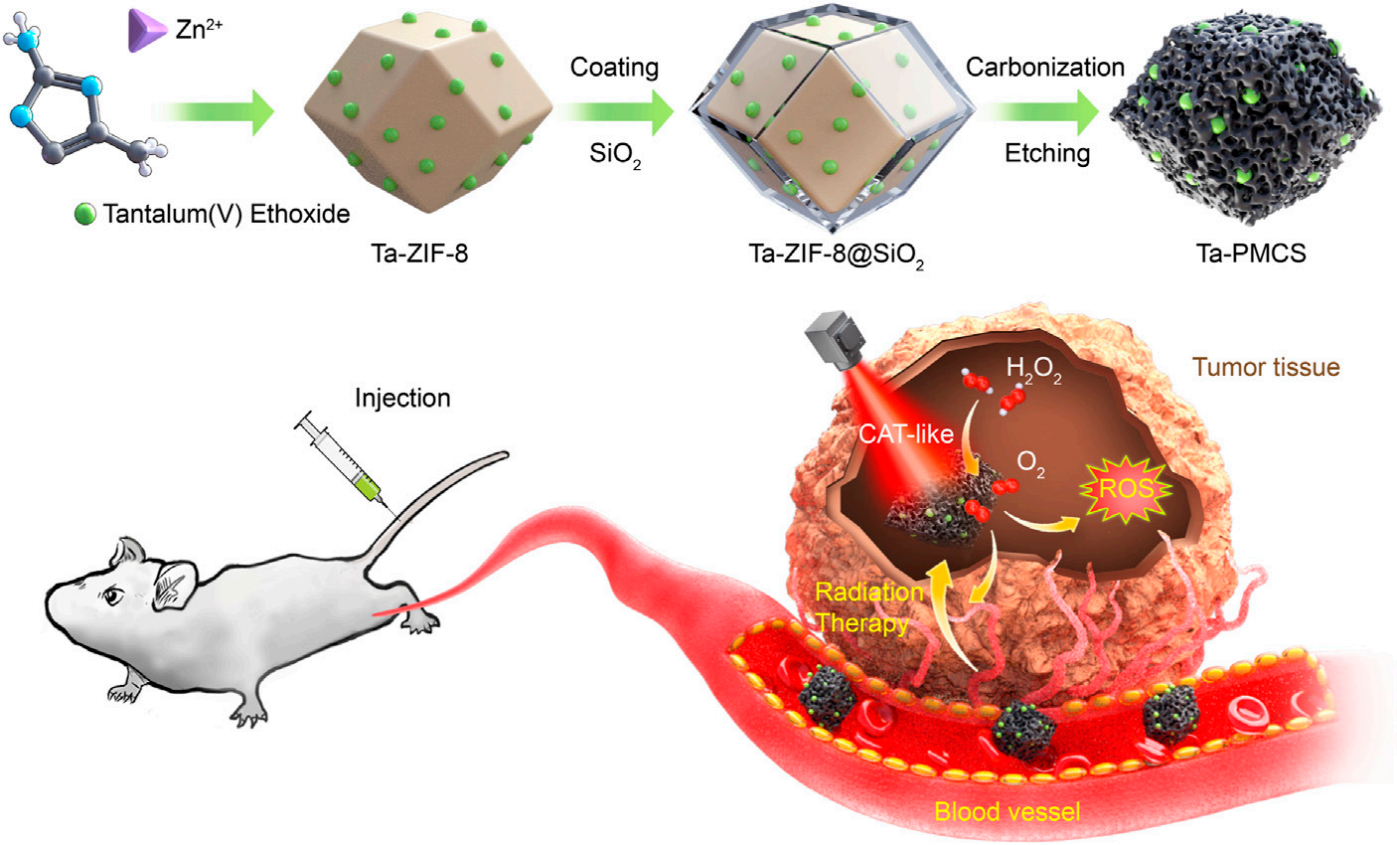
Illustration of the design and synthesis of Ta-PMCS for radiation therapy enhancement.

Tantalum (Ta) is a non-toxic, biologically inert element with a large atomic number (Z = 73) and is a “biophilic metal” with excellent biocompatibility, and it has been widely used in medical implants in the human body (Koshevaya et al., 2021; Mani et al., 2022). According to previous studies, Ta-based nanomaterials can not only be applied as low-toxicity and high-efficiency radiosensitizers but can also be utilised as excellent functional group carriers for loading drugs to modulate the biological behaviour of tumors, which has the advantage of “multi-functional integration” and exhibits considerable potential for biomedical applications (Song et al., 2017b; Chen et al., 2017). In this study, a Ta–carbon nanozyme was used as a logical layout, as shown in Scheme 1, and it demonstrated outstanding catalase-like (CAT-like) activity. It alleviates the hypoxic tumor microenvironment by catalysing hydrogen peroxide and increasing ROS levels to cause more DNA DSBs, thus producing a highly potent anti-tumor effect in the presence of RT. In a human HeLa cancer model, the tumor suppression rate of Ta–carbon nanozyme combined with RT was greater than 81%, which was significantly higher than that of RT alone. In addition, no noticeable side effects of Ta–carbon nanozyme were identified in this study.

Based on these findings, Ta–carbon nanozyme boosted the effectiveness of RT, suggesting that a new treatment strategy was developed to overcome the limitations of RT by combining the enzymatic activity and radiosensitizer properties of nanozymes.

## 2 Materials and methods

### 2.1 Materials

Zn(NO_3_)_2_⋅6H_2_O, 2-methylimidazole, hexadecyl trimethyl ammonium bromide (CTAB), Tantalum(V) ethoxide, and human cervical cancer HeLa cells were acquired from Shanghai Cell Bank and cultured in DMEM medium (Solarbio, China) with 10% foetal bovine serum (FBS, Gibco, United States) and 1% penicillin/streptomycin (Sangon Biotech) in a humidified environment at 37°C and 5% CO_2_. 6-week-old female BALB/c nude mice (17–21 g) were provided by Jiangsu Jicui Yaokang Biotechnology.

### 2.2 Construction of Ta-carbon nanozyme and characterization synthesis of Ta-ZIF-8 nanoparticles

A solution of Zn(NO_3_)_2_⋅6H_2_O (32 mmol in 500 ml CH_3_OH) was poured into a solution containing 135 mmol 2-methylimidazole, tantalum(V) ethoxide, and 0.3 mmol CTAB in methanol (400 ml), and the resulting solution was stirred for 2 h at room temperature. The white solid precipitate was separated *via* centrifugation and washed with methanol.

### 2.3 Synthesis of Ta-zif-8@msio_2_ core@shell nanoparticles

ZIF-8 was dispersed in 240 ml of a 10 vol% methanol solution that had already been adjusted to pH using NaOH; subsequently, 6 ml of an aqueous CTAB (0.068 mol/L) solution was added. Thereafter, TEOS (1.2 ml) was added dropwise to the solution, and the resulting dispersion was stirred for 0.5 h. The resulting core-shell nanoparticles were separated through centrifugation and washed with ethanol.

### 2.4 Synthesis of porphyrin-like mesoporous carbon nanozyme

The Ta-ZIF-8@mSiO_2_ core@shell sample was pyrolysed at 800°C for 2 h under flowing N_2_ and thereafter allowed to cool slowly to room temperature. The pyrolysed sample was subsequently etched with a 4 M NaOH solution to remove the mSiO_2_ shell, followed by centrifugation and washing with deionised water several times until the supernatant was neutral.

### 2.5 Characterization

The morphology of the nanozyme was characterised using transmission electron microscopy (TEM; JEM-2010 ES500W, Japan) and field emission scanning electron microscopy (SEM; Zeiss Merlin Compact). SEM images were captured, and energy-dispersive spectrometry (EDS) mapping was performed using a TESCAN MIRA4 instrument. TEM was performed using a Titan G260-300 field-emission electron microscope. Particle distribution analysis was performed on a Malvern Zetasizer Nano ZS90 instrument. Powder X-ray diffraction patterns were measured on a Bruker D8 Advance 25, and the data were collected in the range of 5–45° at a scan rate of 15 min^−1^. Fourier-transform infrared spectroscopy was performed using a Thermo Scientific Nicolet 6700 spectrometer.

### 2.6 Cell counting kit-8 assay (CCK8 assay)

HeLa cells were seeded into 96-well plates at a density of 4000 cells/well and incubated overnight. Thereafter, the cells were treated with Ta–carbon nanozyme and administered 0 or 6 Gy RT. Cell viability was detected using CCK8 reagent (HY-K0301, MCE), as instructed. Briefly, 10 μL CCK8 reagent with 90 μL serum free medium was added to each well and cultured for 1 h in a 37°C incubator. Optical density values at 450 nm were measured using a microplate reader (ELx800, BioTek, United States).

### 2.7 Colony formation and cell cycle assays

Colony formation assays were used to assess the effectiveness of Ta–carbon nanozyme for radiosensitisation. Cells were evenly seeded into 6-well plates (1000 per well) and incubated for 10–14 days after different treatments. Thereafter, the cells were fixed in methanol for 20 min, stained with crystal violet solution for 15 min, washed in PBS, air-dried, and photographed. Finally, colonies (more than 50 cells) were counted manually using a microscope (0D140BC, Ningbo Shunyu Instrument Co., Ltd.). For cell cycle distribution, cells were collected, fixed overnight in 75% ice-cold ethanol, and incubated with propidium iodide (PI) solution (Seville, Wuhan, China) with RNase for 30 min at 37°C protected from light. The cell cycle distribution was examined using a CytoFLEX flow cytometer (Beckman Coulter, United States) and visualised using Modfit software (Version 3.1).

### 2.8 Transwell migration and invasion assay

Transwell assays were performed to determine the migration and invasion abilities of HeLa cells. Cells in a suspension containing 200 μL of serum-free DMEM medium at a density of 5 × 10^5^/ml were inoculated in Transwell chambers (Corning, NY, United States) with or without matrix gel coating, subsequently placed in 600 μL of DMEM medium with 20% FBS, and incubated for 24 h in a 37°C incubator. Thereafter, the cells were fixed in 4% paraformaldehyde, stained with 0.1% crystalline violet, and the inside of the chambers was wiped off with a swab. Five random fields from each chamber were selected for analysis. The images were photographed and collected using an inverted microscope.

### 2.9 Apoptosis and live/dead assay

To detect the cell apoptosis rate, we used the Annexin V-FITC/PI (#KGA108, KeyGen Biotech, China) double staining method. A total of 106 cells were suspended in 500 μL binding buffer containing 5 μL AnnexinV-FITC and 5 μL PI. The cells were incubated in the dark at room temperature for 15 min and quantified using FACS analysis. The data were analysed using FlowJo software (version 10). To distinguish living cells from dead cells, a Calcein/PI Cell Viability/Cytotoxicity Assay Kit (#C2015M, Beyotime Biotechnology, China) was used to perform the live/dead assay. Cells were incubated with Calcein AM/PI assay solution at 37°C for 30 min and thereafter observed under a fluorescence microscope, where Calcein AM stained live cells with green fluorescence and PI stained dead cells with red fluorescence.

### 2.10 Intracellular reactive oxygen species (ROS) generation

An ROS assay kit (#S0033S, Beyotime, China) using the fluorescent probe DCFH-DA was used to assess the intracellular levels of ROS. The DCFH-DA probe was diluted with 1:1000 in serum-free medium and incubated with cells at 37°C for 30 min before detection of ROS levels through flow cytometry or fluorescence microscopy.

### 2.11 γ-H2AX immunofluorescence analysis

To assess the extent of DNA damage, we performed immunofluorescence analysis to detect phospho-histone-H2AX (γ-H2AX) foci. After RT, the cells were incubated for 2 h and thereafter fixed with 4% paraformaldehyde and Triton X-100 permeated cells. After three washes with PBS, add anti-phosphorylated histone γ-H2AX rabbit monoclonal antibody (#9718, CST, United States) diluted 1:1000 and incubated overnight at 4°C. The next day, the sections were incubated with cy5-conjugated goat anti-rabbit secondary antibody (#GB27303, Servicebio, China) for 1 h at room temperature, stained with DAPI (#C1006, Beyotime, China) for 5 min, and observed under a fluorescent microscope (DMI3000B, Leica, Germany).

### 2.12 *In Vivo* anti-tumor study

All experiments involving mice were approved by the ethics committee of Tongji Hospital. Six-week-old female BALB/c nude mice were purchased from Jiangsu Jicui Yaokang Biotechnology and bred in-house under specific-pathogen-free conditions. HeLa cells (1 × 10^6^/100 μL) were subcutaneously injected into the right lower buttocks of the mice. When the volume of the tumor reached approximately 200 mm^3^, the mice were randomly grouped into four groups (five mice per group): 1) saline; 2) Ta–carbon nanozyme; 3) saline + RT; and 4) Ta–carbon nanozyme + RT. Tumor volume and body weight of the mice were recorded every other day and maintained until the 14th day. Tumor volume was measured as follows: tumor volume (mm^3^) = maximum length (mm) × vertical width (Gong et al., 2021) (mm^2^)/2. At the end of the experiment, orbital blood was extracted from the mice and they were sacrificed through cervical dislocation. Tumor tissue and major tissue organs were removed and fixed with formalin or frozen directly in an 80°C refrigerator to prepare paraffin or frozen sections, respectively.

### 2.13 H&E staining, immunohistochemistry, DHE and HIF-1a

Paraffin sections of tumor tissues were stained with haematoxylin and eosin (H&E) to examine the morphology, structure, apoptosis, and necrosis of the tumor cells. For the proliferation ability of cells, we detected the expression level of Ki-67 through immunohistochemical staining with a Ki67 antibody (#27309-1-AP, 1/400 dilution, Proteintech, China). Dihydroethidium (DHE) and immunofluorescence of HIF-1A were performed to assess oxidative stress and hypoxia levels in tumor tissues.

### 2.14 Biosafety evaluation

To determine the biosafety of Ta–carbon nanozyme, the blood supernatant of mice was collected to detect liver and kidney functions. In addition, paraffin sections of heart, liver, spleen, lung, and kidney were stained with H&E.

### Statistical analysis

All statistical analyses were performed using the GraphPad Prism 8.0.2 software. Data are presented as the mean ± standard deviation. Two-way analysis of variance (ANOVA) or two-tailed Student’s t-test were used to evaluate statistical significance. A *p* value less than 0.05 were regarded as statistically significant (*p < 0.05, **p < 0.01, and ***p < 0.005).

## 3 Results and discussion

### 3.1 Synthesis and characterization of Ta-carbon nanozyme

**FIGURE 1:**
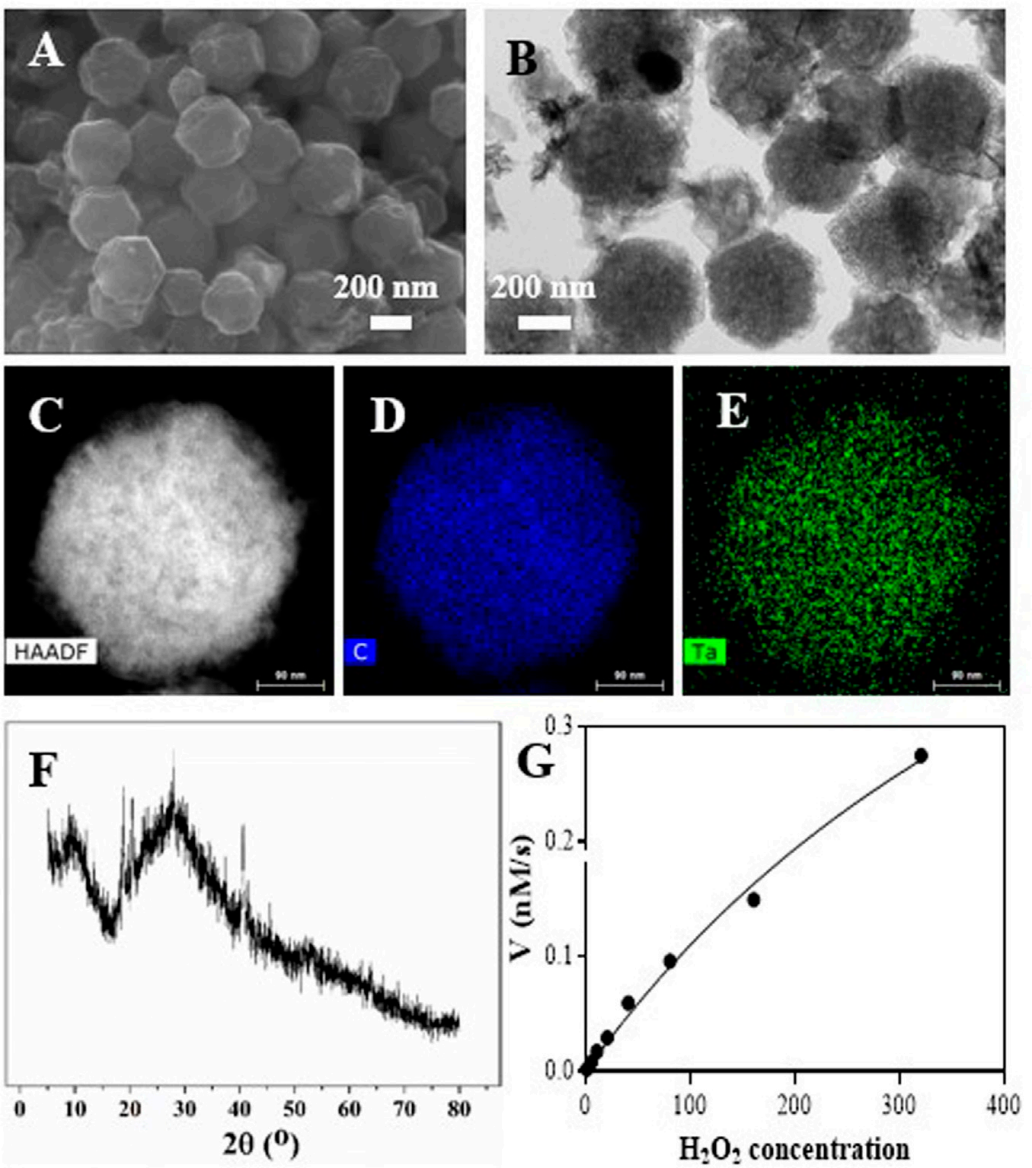
Characterization of the Ta-PMCS. **(A)** SEM image of the Ta-PMCS; **(B)** TEM image of the Ta-PMCS; **(C)** HAADF image of Ta-PMCS; **(D,E)** Elemental mapping images of C, and Ta. **(F)** PXRD of Ta-PMCS; **(G)** catalase-like activity of Ta-PMCS.

The synthesis of Ta–carbon nanozyme is illustrated in Scheme 1. In previous studies, Ta-modified MOF-derived mesoporous carbon nanozymes were first synthesised. The complete synthesis procedure did not involve complex conditions for the steady synthesis of Ta–carbon nanozymes. First, the surface morphology and composition of the Ta–carbon nanozyme were characterised by SEM, TEM, and EDS. As shown in Figure 1A, the prepared Ta–carbon nanozyme had a uniform spherical structure with an average size of approximately 300 nm, which was also confirmed by the TEM image (Figure 1B) and dynamic light scattering (Supplementary Figure S1). The composition distribution was investigated in detail through high-angle annular dark-field scanning TEM mapping, as shown in Figures 1C–E. The EDS data showed that C and Ta were present in the Ta–carbon nanozyme (Supplementary Figure S2). The activity of Ta-carbon nanozyme before and after soaking in water for 14 days indicating its good stability (Supplementary Figure S3). The crystal structure of the Ta–carbon nanozyme was characterised by X-ray diffraction (XRD). The XRD pattern (Figure 1F) exhibited a wide diffraction peak indexed to carbon. After investigating the nanozyme activity, the Ta–carbon nanozyme was observed to have CAT-like activity that can catalyse the decomposition of H_2_O_2_ to generate O_2_ and H_2_O. The amount of O_2_ produced in the system depended on the concentration of H_2_O_2_ in the reaction system. As shown in Figure 1G, with increasing H_2_O_2_ concentration, the rate of oxygen generation also increased.

### 3.2 Ta-carbon nanozyme exerts radiosensitization *in vitro*

**FIGURE 2:**
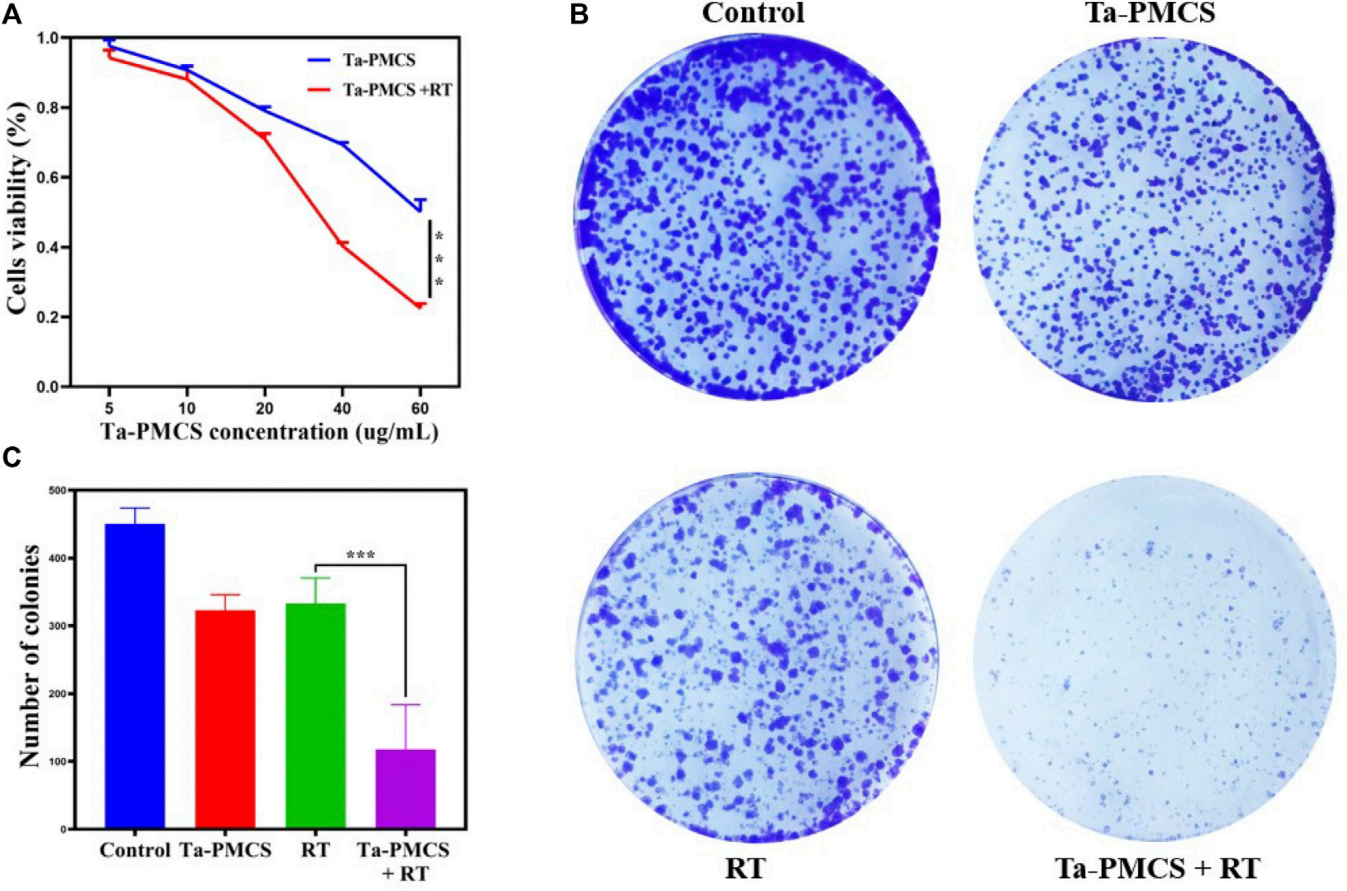
Assessment of the anti-proliferation ability of Ta-PMCS *in vitro*. **(A)** CCK-8 assay of HeLa cells treat with Ta-PMCS or Ta-PMCS + RT at different concentration gradients. **(B,C)** Colony formation assays were used to detect the proliferation of HeLa cells treated with the control, Ta-PMCS, RT or Ta-PMCS + RT. *p < 0.05, **p < 0.01, ***p < 0.005.

**FIGURE 3:**
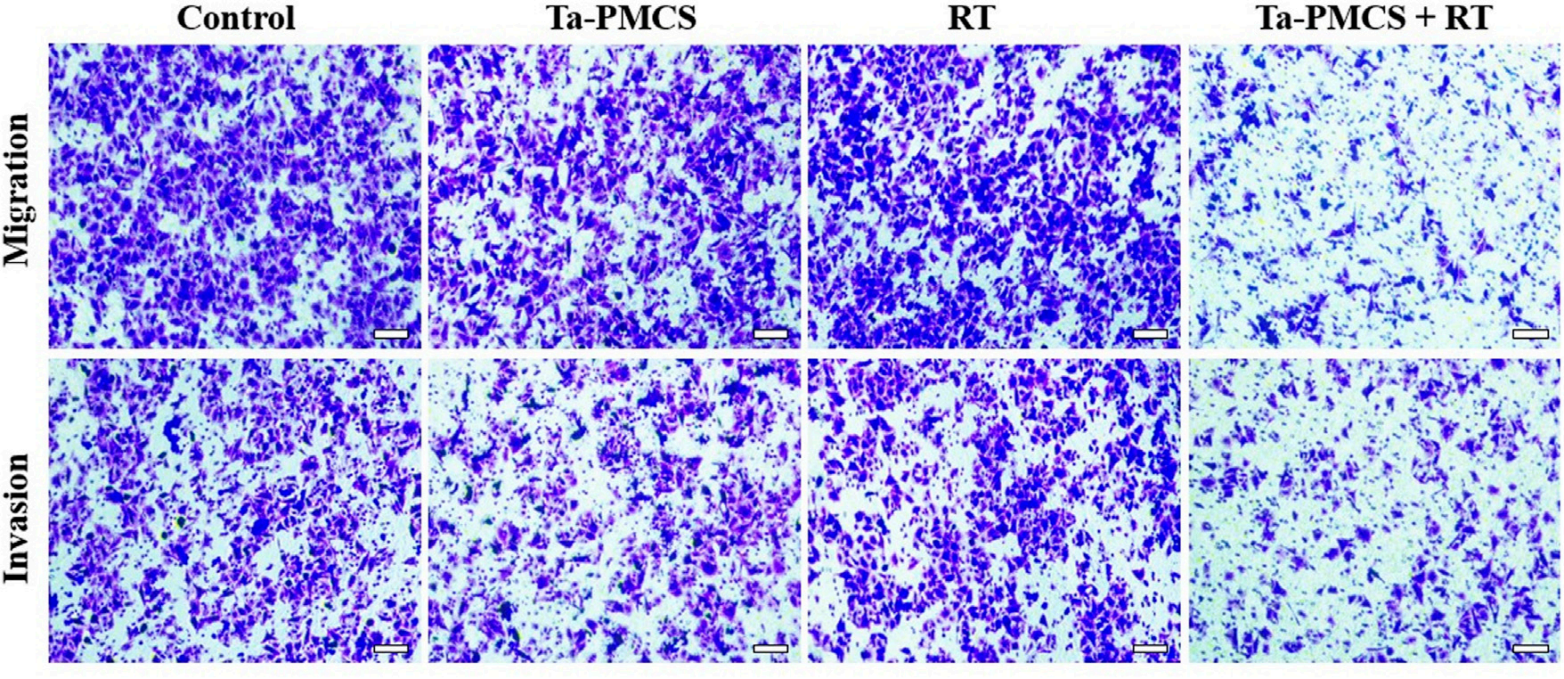
The migration and invasion ability of HeLa cells treated with the control, Ta-PMCS, RT or Ta-PMCS + RT were detected by Transwell assay (scale bar: 50 μm).

**FIGURE 4:**
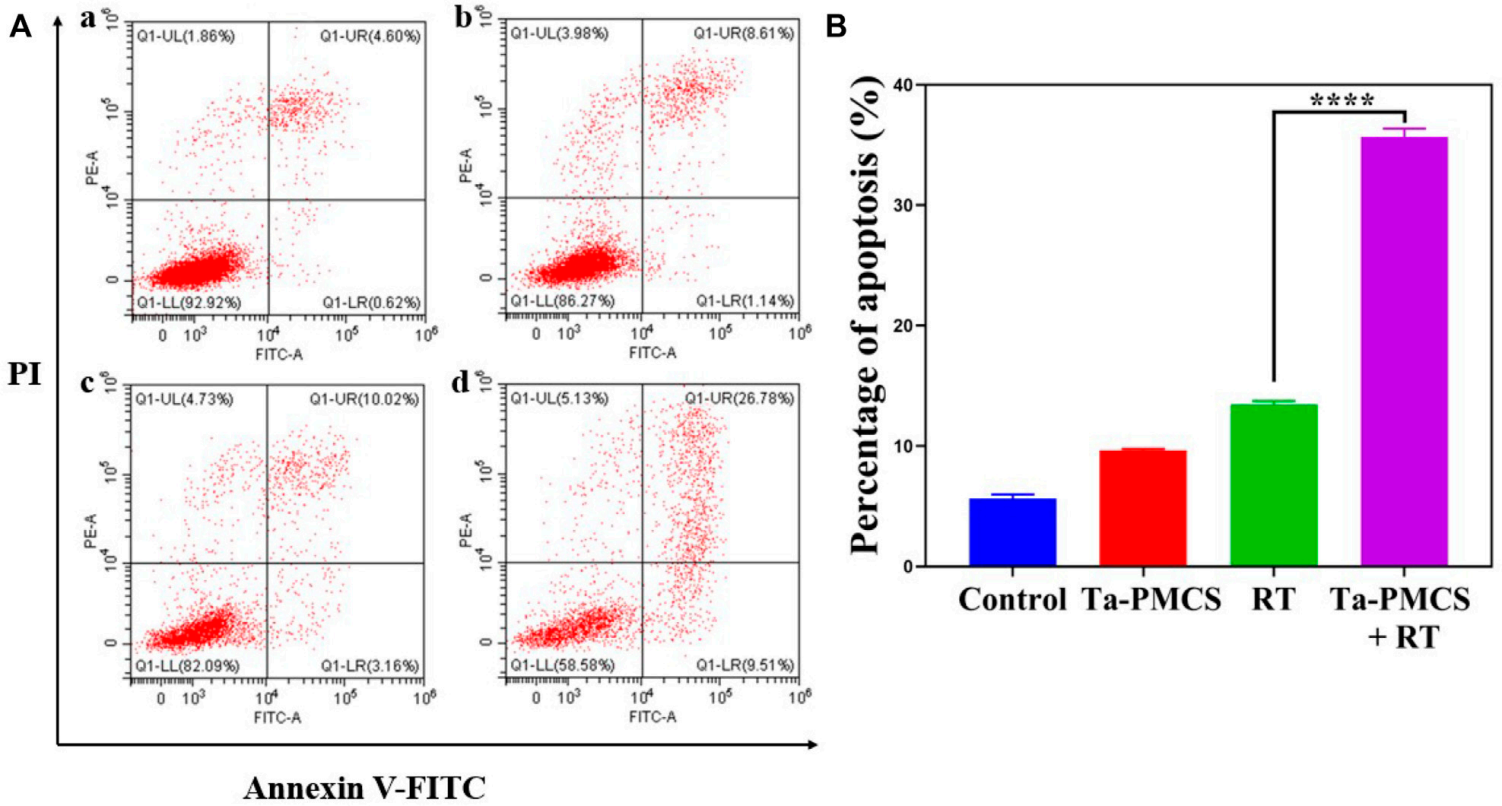
Annexin V-FITC/PI dual-staining was used to assess the apoptosis rates of HeLa cells. **(A)** Control; **(B)** Ta-PMSC (20 ug/mL); **(C)** RT (6 gy); **(D)** Ta-PMSC (20 ug/mL) + RT (6 gy). *p < 0.05, **p < 0.01, ***p < 0.005.

**FIGURE 5:**
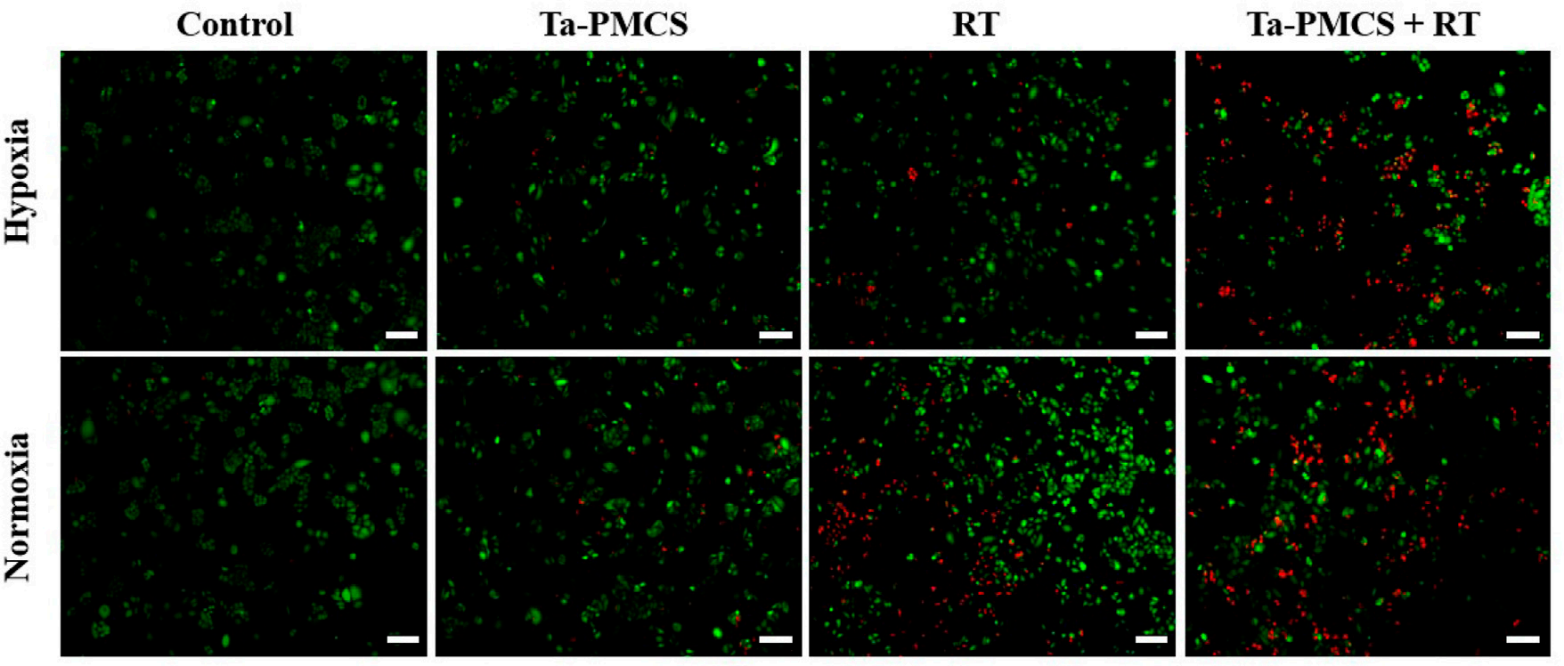
Fluorescence images of HeLa cells co-stained with Calcein AM (live cells, green) and PI (dead cells, red) treat with the control, Ta-PMCS, RT or Ta-PMCS + RT under hypoxia (1% O2) and normoxia (21% O2) conditions (scale bar: 50 μm).

**FIGURE 6:**
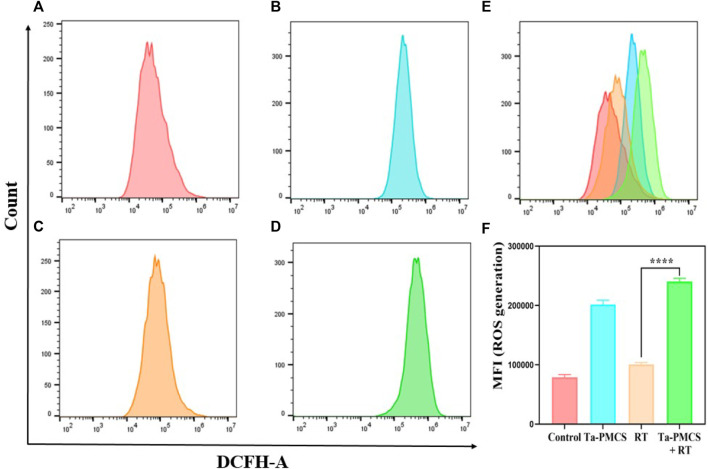
ROS levels were measured by flow cytometry after treat with different treatments. **(A)** Control; **(B)** Ta-PMSC; **(C)** RT; **(D)** Ta-PMSC + RT; **(E)** merged imaged of **(A)**, **(B)**, **(C)** and **(D)**. **(F)** Mean fluorescence intensity of ROS generation. *p < 0.05, **p < 0.01, ***p < 0.005.

**FIGURE 7:**
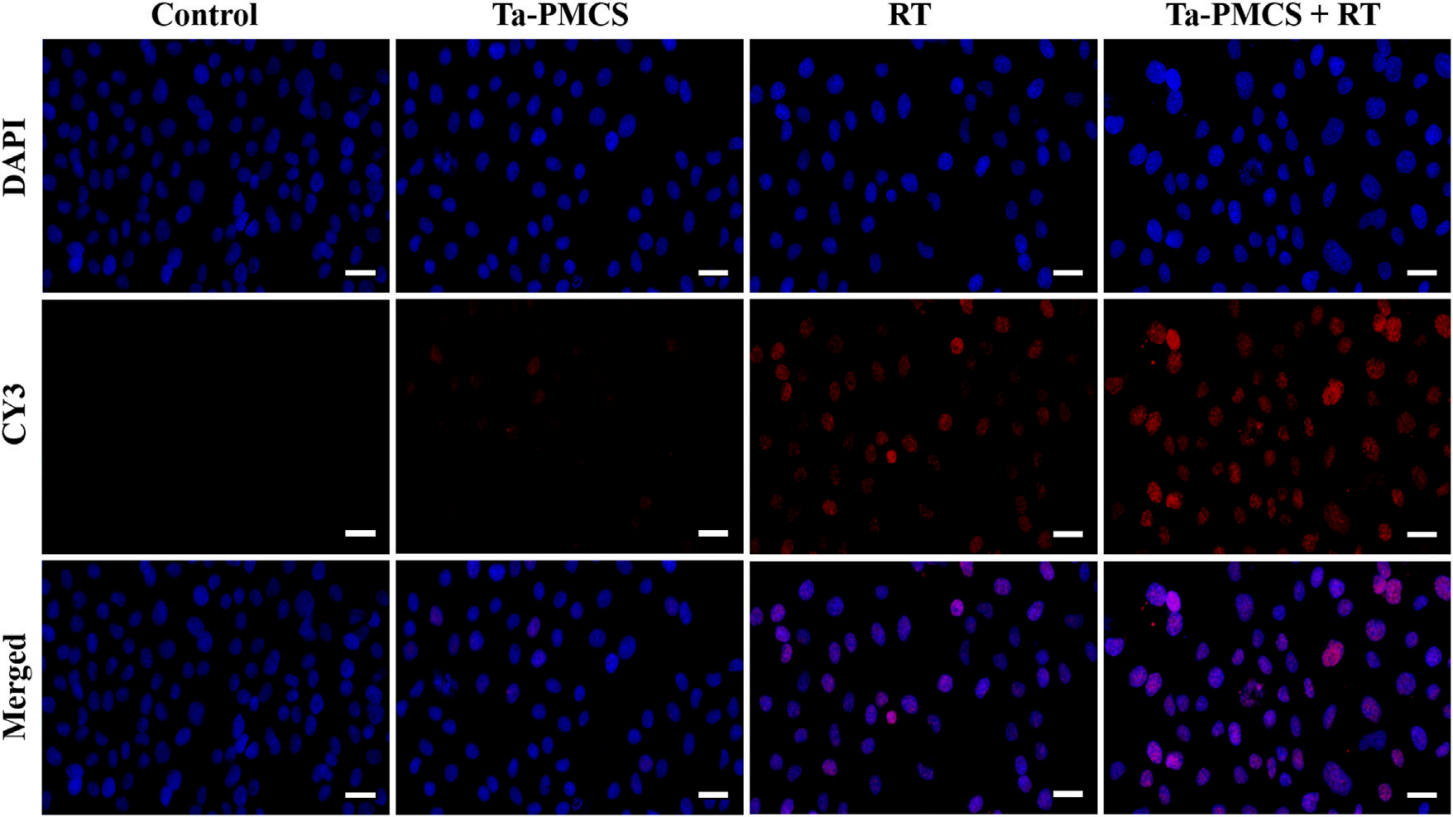
Immunofluorescence staining of γ-H2AX of HeLa cells treated with the control, Ta-PMCS, RT or Ta-PMCS + RT to assess DNA damage (scale bar: 50 μm).

The CCK-8 assay detected the cell viability of Ta–carbon nanozyme at 0 and 6 Gy with different concentration gradients (Figure 2A). When the Ta–carbon nanozyme concentration reached 60 μg/ml, the cell survival rate decreased to approximately 50%, whereas only 22% of the Ta–carbon nanozyme combined with the RT group. These results suggest that the exposure of HeLa cells to Ta–carbon nanozyme following combined RT can significantly reduce the proliferative activity of the cells. Thereafter, we used a concentration of 30 μg/ml of Ta–carbon nanozyme and 6 Gy as the radiation dose for subsequent experimental validation. Meanwhile, the clone formation assays (Figure 2B) indicated that pretreatment with Ta–carbon nanozyme combined with RT significantly inhibited proliferation and colony formation, with an inhibition rate of 74%, which was 26% and 28% in the RT and Ta–carbon nanozyme groups, respectively (Figure 2C). These results support the view that Ta–carbon nanozyme combined with RT has a greater inhibitory effect on HeLa cell proliferation than either Ta–carbon nanoparticles or irradiation alone, indicating that Ta–carbon nanozyme and RT can both suppress cell proliferation and have a synergistic effect. In addition, given that the capacity of tumor cells to migrate and invade is closely related to cancer progression, metastasis, recurrence, and poor prognosis, we conducted transwell experiments to assess the effect of Ta–carbon nanozyme combined with RT on the invasive and migratory capacity of HeLa cells. As anticipated, compared with exposure to Ta–carbon nanozyme or RT alone, after treatment with Ta–carbon nanozyme and RT, the ability of HeLa cells to migrate and invade was significantly weakened (Figure 3). To assess the anti-tumor effect, the apoptosis rate and cell cycle arrest induced by the control, Ta–carbon nanozyme, RT, and Ta–carbon nanozyme + RT were evaluated through flow cytometry. Apoptosis is a key mechanism underlying anticancer effects and radiosensitivity in tumor therapy. In Figures 4A,B, the apoptosis results and apoptosis rates of the different treatment groups are illustrated. When the cells were exposed to Ta–carbon nanozyme and RT separately, the apoptosis rates were 9.75 and 13.18%, respectively. When the two were used in combination, the apoptosis rate increased further and reached 36.29%, indicating that the Ta–carbon nanozyme has a RT-enhancing effect. The cell cycle distribution of the different treatment groups is shown in Supplementary Figure S4. The Ta–carbon nanozyme alone did not block the cell cycle in the G2/M phase before radiation treatment. In comparison to the RT group, Ta–carbon nanozyme combined with RT slightly arrested the cell cycle in the G2/M phase, and it is widely believed that blockade in the G2/M phase triggers the apoptotic cell death. To further evaluate the therapeutic effect of Ta–carbon nanozyme under normoxic and hypoxic conditions, calcein-AM (live cells) and PI (dead cells) staining was performed on HeLa cells. Under normoxia, green fluorescence was observed in almost the entire field of vision, with nearly no cell death in the control group. Under hypoxic conditions, the tumor cell-killing ability of the RT group was weaker than that of the normoxic group. However, the killing ability of the Ta–carbon nanozyme + RT group was comparable to that of the normoxic state, indicating that the Ta–carbon nanozyme has a CAT-like catalytic ability to alleviate the hypoxic state of tumor cells (Figure 5). To understand the sensitisation mechanism of RT in depth, ROS and γ-H2AX levels were measured. ROS are mainly derived from mitochondria and contribute to the level of oxidative stress in cells. Previous studies have reported that ROS can damage proteins, cause DNA damage, and induce apoptosis. Thus, we measured ROS levels in each group, as shown in Figure 6. According to the FCM results, intracellular ROS levels were elevated in cells treated with Ta–carbon nanozyme alone, 2.5 times higher than in the control group and 2 times higher than in the RT alone treatment group. When Ta–carbon nanozyme was combined with RT, ROS levels were 2.4 times higher than those in the RT group. Fluorescence microscopy images confirmed this change in the ROS levels (Supplementary Figure S5). This implies that more DNA DSBs can be generated, resulting in a sensitising effect of RT. The generation of DSBs in cancer cells offers a deeper understanding of radiation-induced lesion development within these cells, and the measurement of γ-H2AX levels is a highly sensitive tool for identifying DSB generation in response to irradiation. Therefore, nuclear γ-H2AX foci were measured through immunofluorescence staining with different treatment groups (Figure 7). Cells treated with Ta–carbon nanozyme + RT demonstrated a significant increase in the number of DSBs compared to cells treated with Ta–carbon nanozyme or RT alone. The observed increase in DNA damage may be due to cellular uptake of Ta nanoparticles, increasing the dose of RT precipitation, and relieving intracellular hypoxia in tumor cells, leading to the formation of more γ-H2AX lesion. The quantitative assessment of γ-H2AX foci density revealed that the γ-H2AX foci levels were 1.6-fold higher in the Ta–carbon nanozyme + RT treatment group than in the RT treatment group, illustrating the potential of Ta–carbon nanozyme as an effective radiosensitizer.

### 3.3 Tantalum-carbon nanozyme composites inhibited tumor growth *in vivo*

**FIGURE 8:**
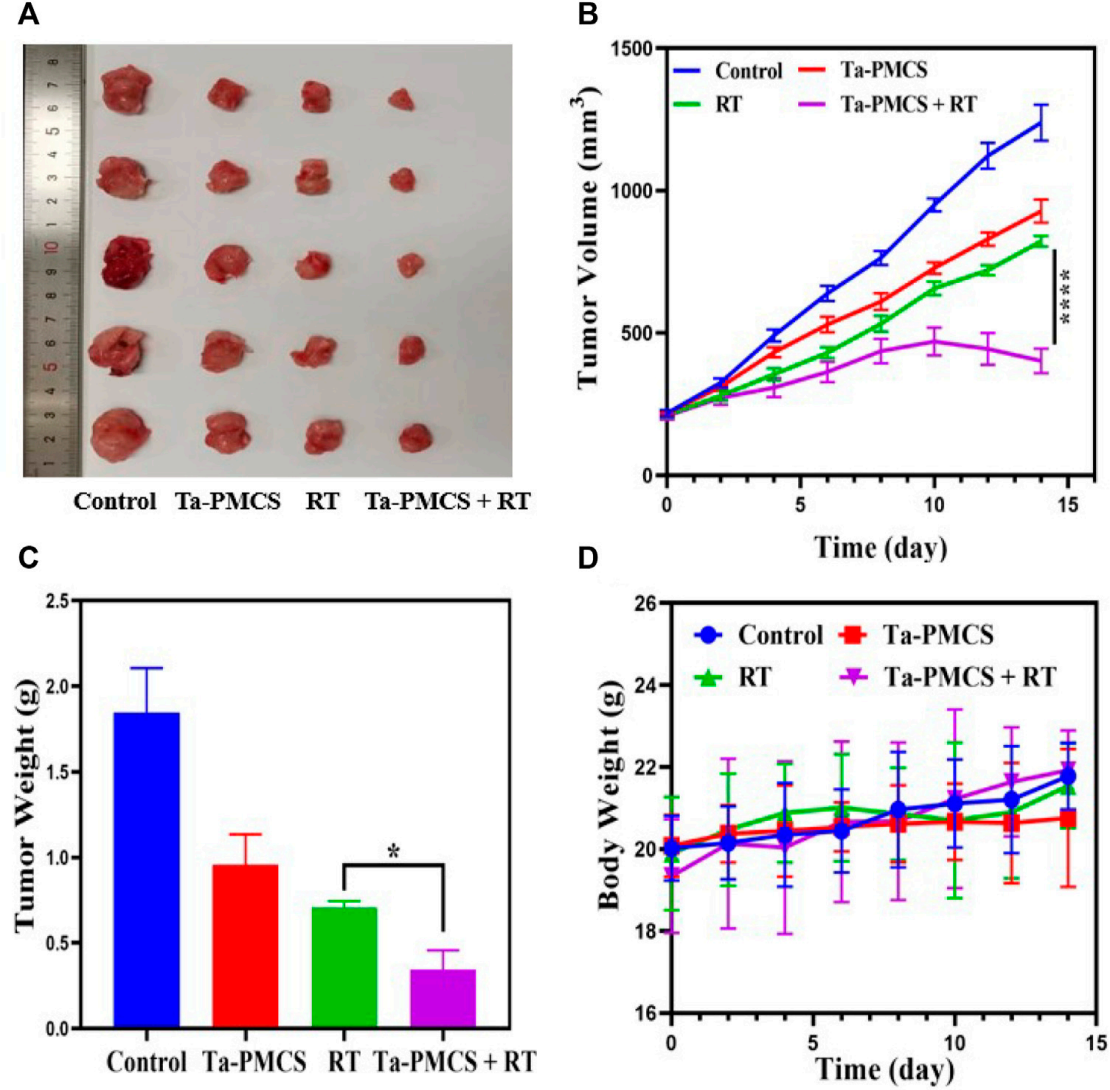
Tumor growth inhibition after treated with the control, Ta-PMCS, RT or Ta-PMCS + RT *in vivo*. **(A)** The gross tumor appearance after treat with different treatments; **(B)** tumor volume changes in different groups; **(C)** tumor weight; **(D)** body weight changes. *p < 0.05, **p < 0.01, ***p < 0.005.

**FIGURE 9:**
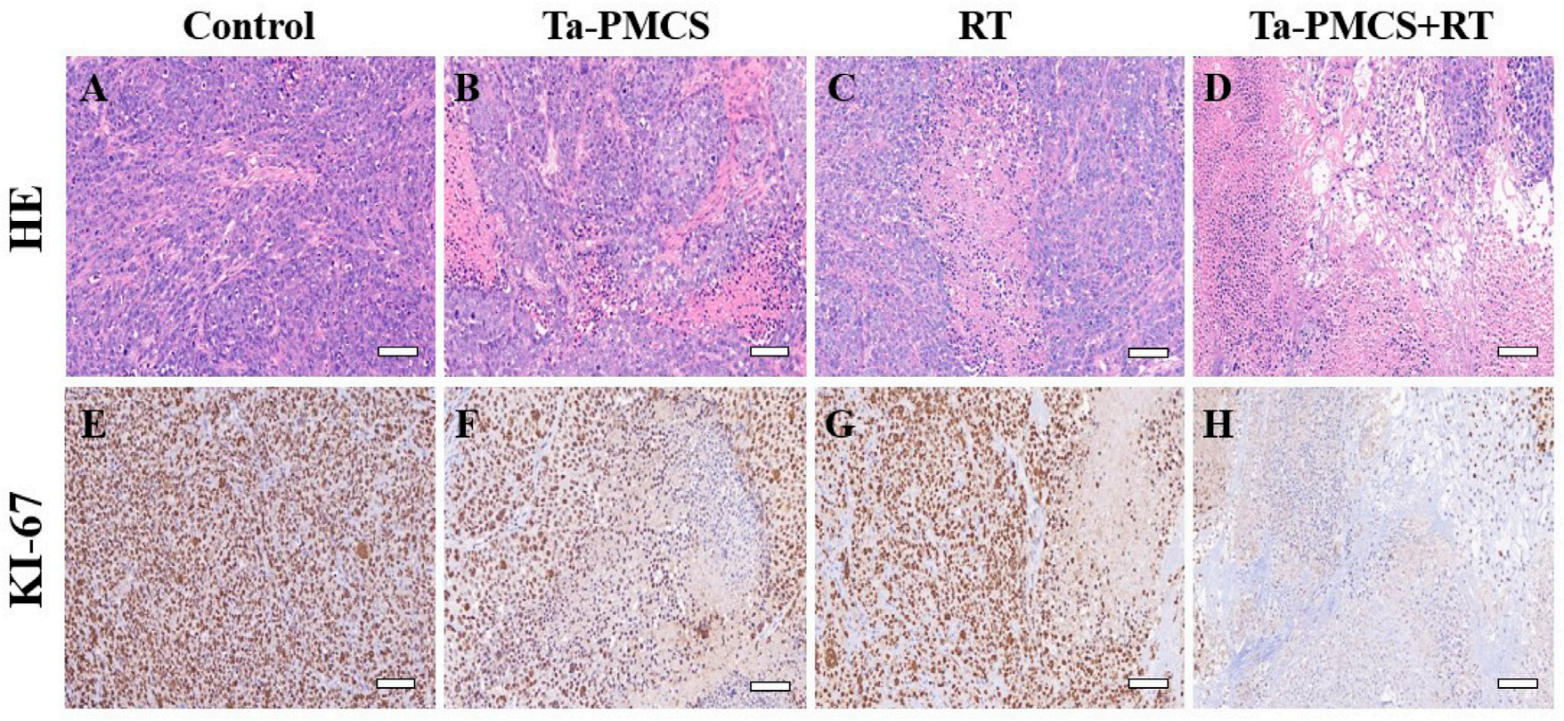
HE and Ki67 staining of tumor sections in different groups (scale bar: 50 μm). **(A,E)** Control; **(B,F)** Ta-PMCS; **(C,G)** RT; **(D,H)** Ta-PMCS+RT.

**FIGURE 10:**
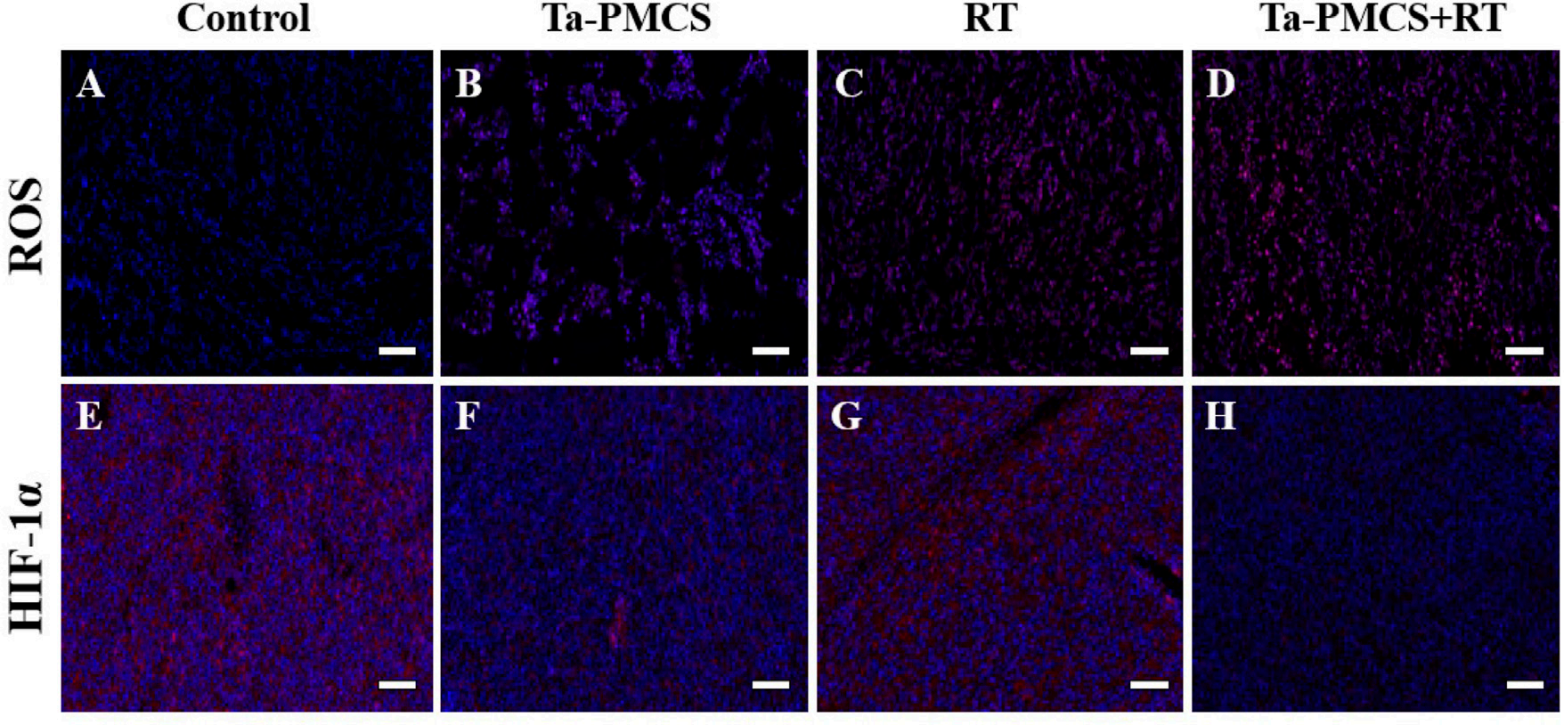
ROS and HIF-1α staining of tumor sections in different groups (scale bar: 50 μm). **(A,E)** Control; **(B,F)** Ta-PMCS; **(C,G)** RT; **(D,H)** Ta-PMCS+RT.

**FIGURE 11:**
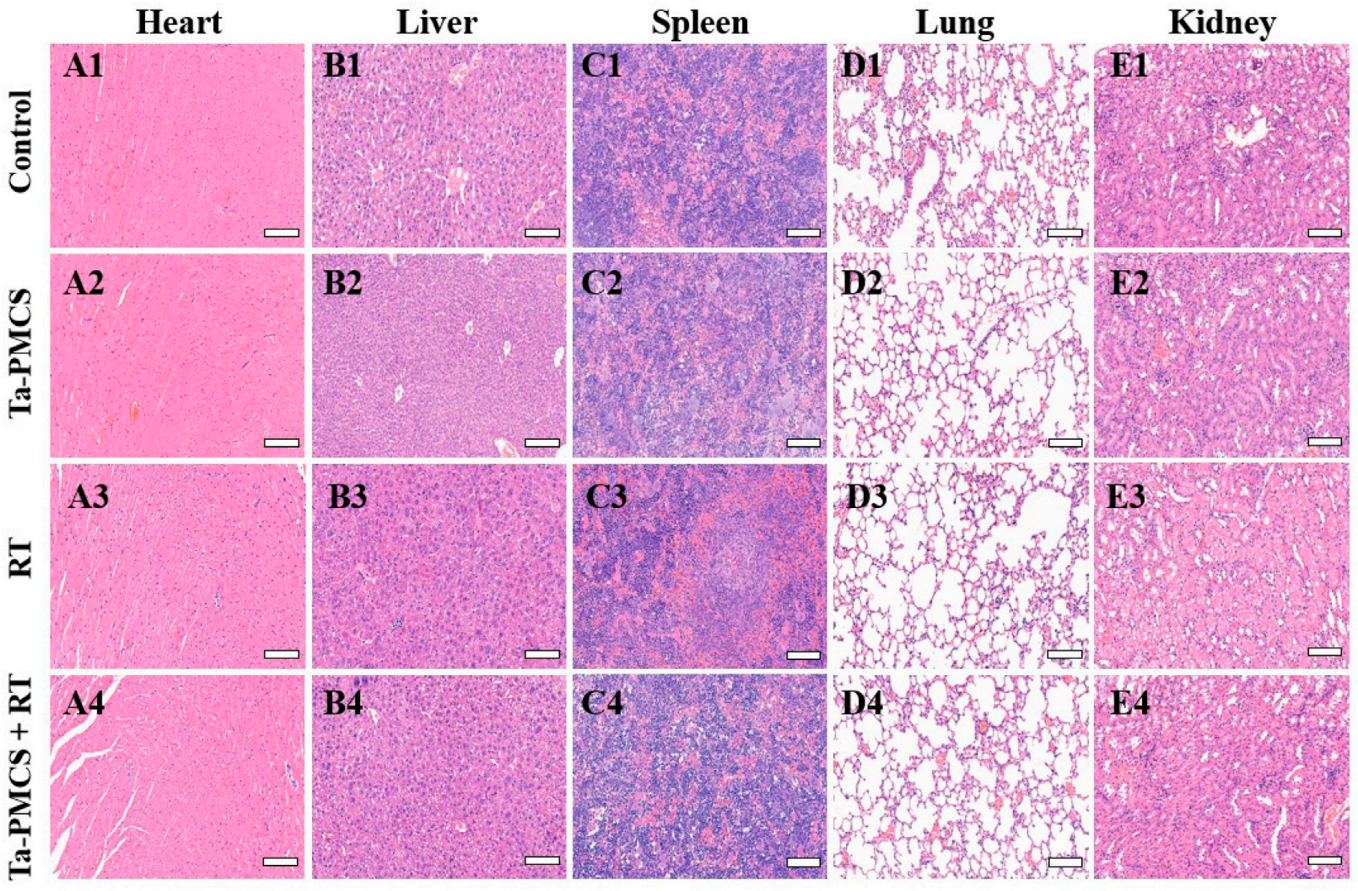
HE staining of major organs in mice injected with different treatments (scale bar: 50 μm).

Considering the biological properties of Ta–carbon nanozyme, such as favourable anti-tumor effects *in vitro* by increasing ROS levels and alleviating hypoxia in the tumor microenvironment, we further investigated the inhibitory effect and biosafety of Ta–carbon nanozyme in a HeLa cancer-bearing mouse model. A total of 10^6^ cells were subcutaneously injected into the mice to construct HeLa tumor-bearing BALB/c nude mice. At the time point when the tumor volume was 200 mm^3^, the mice were divided into four groups (n = 4): 1) saline solution group, 2) Ta–carbon nanozyme group (8 mg/kg), 3) RT group (6 Gy), and 4) Ta–carbon nanozyme + RT group (8 mg/kg, 6 Gy). The mice were administered saline or Ta–carbon nanozyme *via* the tail vein at a dose of 8 mg/kg once every other day three times, followed by 0 or 6 Gy RT 24 h later, respectively. Tumor volume and body weight were measured every alternate day for each group of mice. Thereafter, the mice were sacrificed on day 14, and the tumors were weighed simultaneously. The gross appearance of the tumor is shown in Figure 8A. As shown in Figures 8B,C, the tumor volume in the saline group (control group) increased rapidly. The Ta–carbon nanozyme and RT alone group exhibited a certain tumor inhibition effect, and the Ta–carbon nanozyme + RT group exhibited the best tumor suppression effect (82%), suggesting that Ta–carbon nanozyme can fully improve the efficiency of RT. There was no severe body weight loss during the observation period among the four groups, indicating minimal systemic toxicity and no significant side effects (Figure 8D). Thereafter, H&E, Ki-67, ROS, and HIF-1a staining were collected from each group of tumor sections. H&E (Figures 9A–D) staining revealed that the Ta–carbon nanozyme combined with RT induced a significant reduction in the number of tumor cells and an increase in the rate of apoptosis or necrosis compared with other treatment modalities. In Figures 9E–H, immunohistochemistry Ki-67 staining results confirmed that the Ta–carbon nanozyme + RT group was the most effective in inhibiting proliferation. We then assessed the oxidative stress and hypoxia levels in the tumor tissue. Measurement of intratumoral ROS generation in mice treated with the DCF exhibited significantly enhanced staining in mice that had been treated with the Ta–carbon nanozyme + RT combination (Figures 10A–D). Because the neoplastic microenvironment in solid tumors is characterised by a typical hypoxic state, it promotes tumor progression and resistance to RT, and HIF‐1α is an important regulator of hypoxia. Therefore, we assessed the expression levels of HIF-1α in tumor tissues using immunofluorescence staining. In comparison with the control group, HIF expression was reduced in the Ta–carbon nanozyme group, indicating that the Ta–carbon nanozyme exhibits peroxidase activity, alleviates hypoxia in the tumor microenvironment, and has the potential for RT sensitisation (Figures 10E–H). These results suggest that the combination of Ta–carbon nanozyme and RT effectively overcomes intratumor hypoxia and enhances oxidative stress levels, thereby enhancing DNA DBS and antitumor effects. In recent years, the excellent performance of nanomaterials in drug delivery, molecular imaging, tumor therapy, and several other applications makes them a promising biomedical application material. The underlying toxicity *in vivo* is a considerable concern for the use of nanomaterials in pharmaceutical and clinical applications. Thus, at the end of this study, we evaluated the biosafety of the Ta–carbon nanozyme in mice. As shown in Figure 11, the tissue structure and cell morphology of the main organs (heart, liver, spleen, lung, and kidney) stained by H&E confirmed that these nanoparticles had little or no systemic toxicity when administered *in vivo*. Moreover, the plasma biochemical indices of the different treatment groups were within the normal range, and serum aspartate aminotransferase, alanine aminotransferase, alkaline phosphatase, creatinine, and blood urea nitrogen of the mice did not exhibit any significant abnormalities, implying that the liver and kidney functions were normal (Supplementary Figure S6). The obtained results suggest that the Ta–carbon nanozyme exhibits excellent biocompatibility and may offer a promising strategy for RT.

## 4 Conclusion

In this study, we successfully constructed a viable and efficient Ta–carbon nanozyme with prominent CAT-like activity and ROS amplification to enhance tumor RT by alleviating hypoxia and generating ROS. The synthesis of Ta–carbon nanozymes is simple, convenient, and low-cost, without the requirement of multiple enzymes and complicated reactions. It exhibits CAT-like activity and catalyses the decomposition of H_2_O_2_ to generate O_2_ and H_2_O, improving the production of oxygen in the tumor microenvironment. Additionally, with a higher atomic number of TA, its absorption capacity for X-rays is strong, which can increase the dose of radiation precipitation, and the ROS produced can cause more DNA DSBs, thereby enhancing the effect of RT. In a human cervical cancer model, the tumor suppression rate of the Ta–carbon nanozyme combined with the RT group was more than 80%, which was markedly higher than that of RT alone group (62%). In addition, H&E staining of important organs and liver and kidney functions demonstrated that the Ta–carbon nanozyme had no clear toxic side effects *in vivo*. Therefore, this study provides a simple, feasible, clinically valuable, safe, and effective RT treatment strategy for overcoming RT resistance.

## Data Availability

The original contributions presented in the study are included in the article/Supplementary Material, further inquiries can be directed to the corresponding authors.
