# Supplementary material for: Tantalum-carbon-integrated nanozymes as a nano-radiosensitizer for radiotherapy enhancement
Source: Front Bioeng Biotechnol. 2022 Oct 24;10:1042646. doi: 10.3389/fbioe.2022.1042646 (PMC9638097; doi:10.3389/fbioe.2022.1042646)
Supplement: Supplementary file 1 [file DataSheet1.docx]

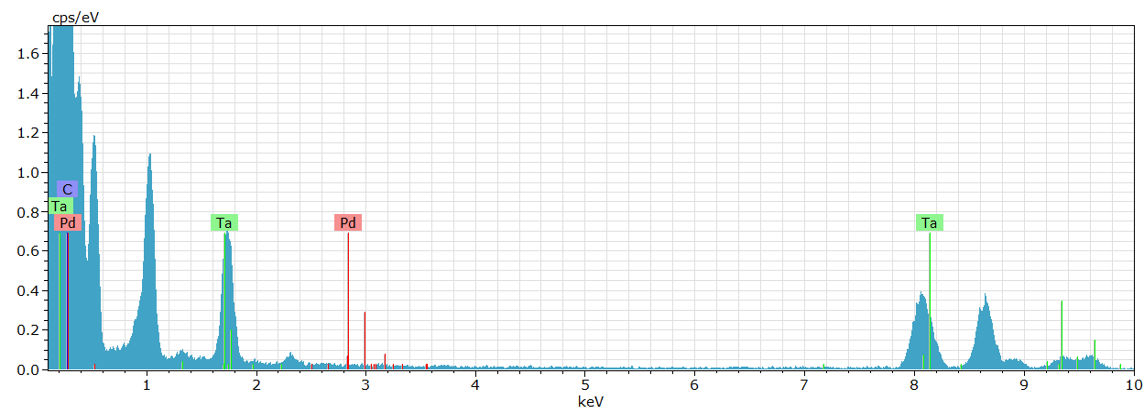


**Fig. S1** EDS of Ta-PMCS.


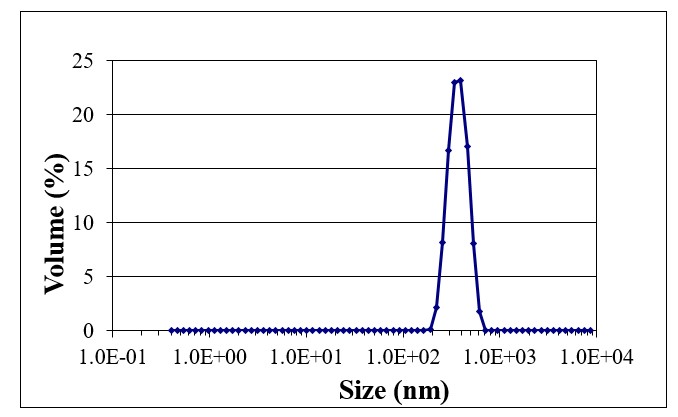


**Fig. S2** DLS of Ta-PMCS.


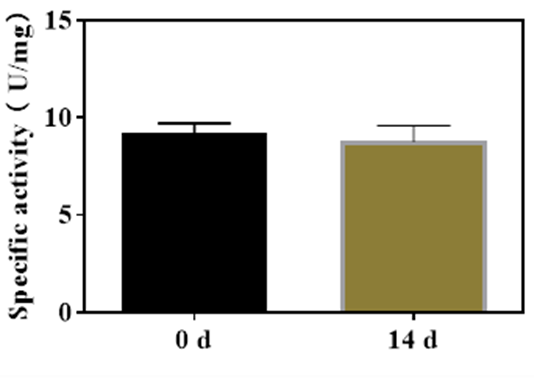


Fig S3. The activity of Ta-carbon nanozyme before and after soaking in water for 14 d.


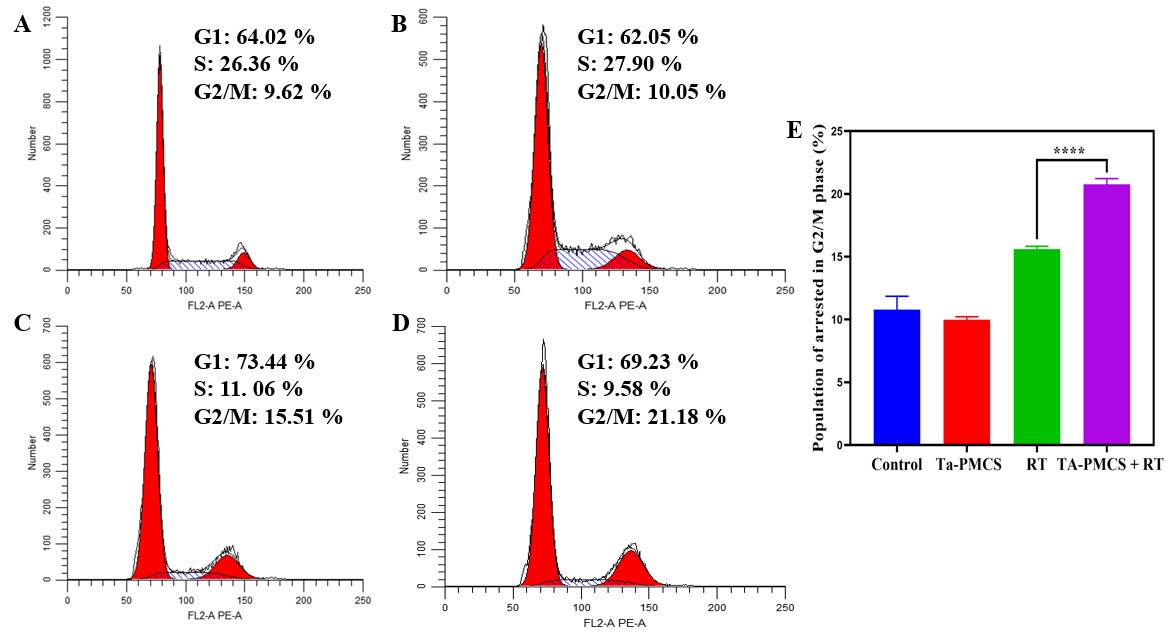


**Fig. S4** Cell-cycle distribution in different treatment groups. *P < 0.05, **P < 0.01, ***P < 0.005.


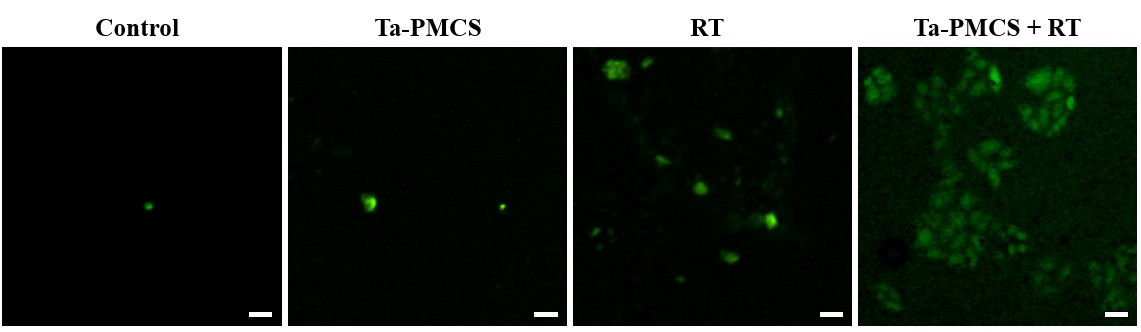


**Fig. S5** ROS levels in Hela cells treated with treated with the control, Ta-PMCS, RT or Ta-PMCS + RT (scale bar: 50 μm).


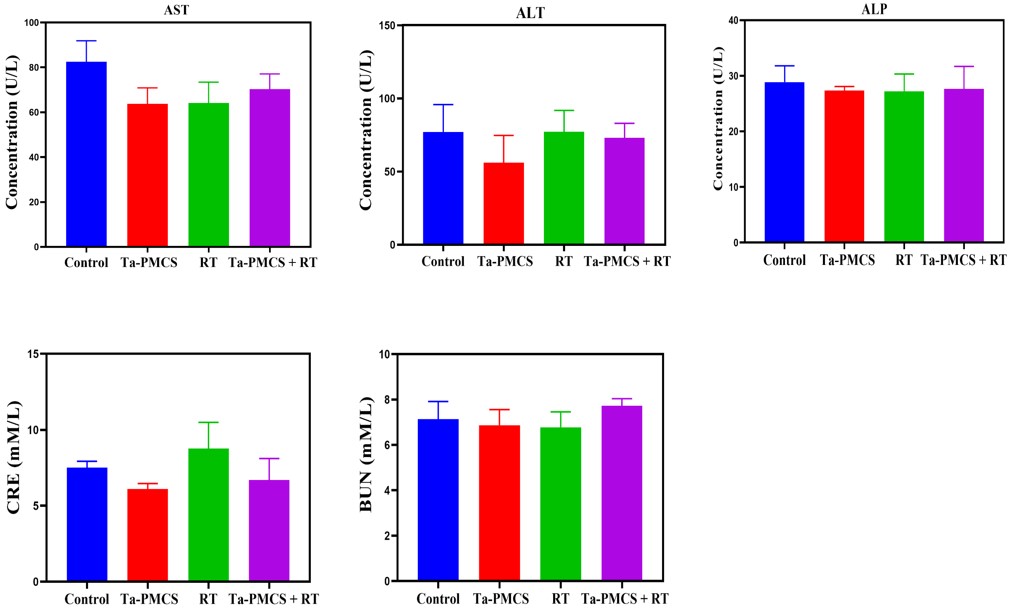


**Fig. S6** Blood biochemistry data of liver function markers (AST, ALT, and ALP) and kidney function markers (CRE, and BUN).
